# Protective Effect of Quercetin against Oxidative Stress-Induced Cytotoxicity in Rat Pheochromocytoma (PC-12) Cells

**DOI:** 10.3390/molecules22071122

**Published:** 2017-07-06

**Authors:** Dengke Bao, Jingkai Wang, Xiaobin Pang, Hongliang Liu

**Affiliations:** 1 Institute of Pharmacy, Pharmaceutical College of Henan University, Kaifeng 475004, China; 10200102@vip.henu.edu.cn (D.B.); hndxpxb@163.com (X.P.); 2 Department of Nursing, NanYang Medical College, NanYang 473000, China; wjk315zjy329@163.com

**Keywords:** quercetin, antioxidant, oxidative stress, Parkinson’s disease

## Abstract

Oxidative stress has been implicated in the pathogenesis of many kinds of neurodegenerative disorders, particularly Parkinson’s disease. Quercetin is a bioflavonoid found ubiquitously in fruits and vegetables, and has antioxidative activity. However, the underlying mechanism of the antioxidative effect of quercetin in neurodegenerative diseases has not been well explored. Here, we investigated the antioxidative effect and underlying molecular mechanisms of quercetin on PC-12 cells. We found that PC-12 cells pretreated with quercetin exhibited an increased cell viability and reduced lactate dehydrogenase (LDH) release when exposed to hydrogen peroxide (H_2_O_2_). The significantly-alleviated intracellular reactive oxygen species (ROS), malondialdehyde (MDA), and lipoperoxidation of the cell membrane of PC-12 cells induced by H_2_O_2_ were observed in the quercetin pretreated group. Furthermore, quercetin pretreatment markedly reduced the apoptosis of PC-12 cells and hippocampal neurons. The inductions of antioxidant enzyme catalase (CAT), superoxide dismutase (SOD), and glutathione peroxidase (GSH-Px) in PC-12 cells exposed to H_2_O_2_ were significantly reduced by preatment with quercetin. In addition, quercetin pretreatment significantly increased Bcl-2 expression, and reduced Bax, cleaved caspase-3 and p53 expressions. In conclusion, this study demonstrated that quercetin exhibited a protective effect against oxidative stress-induced apoptosis in PC-12 cells. Our findings suggested that quercetin may be developed as a novel therapeutic agent for neurodegenerative diseases induced by oxidative stress.

## 1. Introduction

The balance between oxidant and anti-oxidant intracellular systems is very important for cell function. Disruption in the antioxidant redox system results in excessive production and progressive accumulation of reactive oxygen species (ROS), such as super oxygen ions, H_2_O_2_ and hydroxy free radicals, etc. ROS produced during normal metabolism of oxygen plays an important role in cell signaling [1]. However, excessive ROS production during various pathological conditions is a potent inducer of apoptosis, which causes oxidative damage to various biological macromolecules, including DNA, proteins in plasma, and lipids in cell membranes, thereby disrupting cellular function and initiating subsequent cell death via necrosis or apoptosis [2,3,4]. Many studies show that oxidative damage by ROS is widely implicated in neurodegenerative disorders. Formation of ROS has been proposed to be a vital step leading to neuronal death related to neurodegenerative diseases, such as Alzheimer’s disease (AD) and Parkinson’s disease (PD) [5,6]. Therefore, alleviating or preventing oxidative damage by ROS should be a potential therapeutic target for neurodegenerative diseases.

Quercetin (3,3′,4′,5,7-hydroxy flavone) is an anti-oxidative bioflavonoid distributed widely in herbs, vegetables, and fruits used in human diets [7,8]. Quercetin has been demonstrated to modulate several signaling pathways, such as Nrf2/keap1, NF-κB, and MEK/ERK, which are involved in the processes of carcinogenesis and inflammation [9]. Quercetin has been reported to possess anticancer effects through suppressing the oncogene expression, such as *c-fos*, *c-jun*, and *ras* [10,11,12]. Quercetin can also relieve inflammation through inhibiting NF-κB signal and TNF-α production, which is induced by lipopolysaccharide (LPS) [13,14,15,16]. Moreover, it was reported that quercetin is a potential candidate against oxidative stress-induced organ damage [17]. Although several studies have demonstrated the antioxidant property of quercetin [18,19,20,21], but the neuroprotective effects of quercetin is not well explored and the antioxidant molecular mechanisms remain obscure. In this study, we investigated the neuroprotective effects of quercetin on H_2_O_2_-induced apoptosis in PC-12 cells and hippocampal slices as well as elucidated the antioxidant mechanisms of quercetin.

Rat pheochromocytoma, PC-12 cell line, is commonly used in the investigation of neurotherapeutics study for neurodegenerative diseases, such as AD and PD [22,23]. The PC-12 cell line is also well known to secrete the neurotransmitter dopamine, resemble dopaminergic cells, and possess dopamine transporters. Compared with the primary cultured neurons and PC-12 cells, hippocampal slices maintain a morphology and connectivity that are similar to native brain tissue, which was well documented for morphological identification of nerve cells in physiological and pharmacological studies [24,25].

## 2. Results

### 2.1. Dose Response of H_2_O_2_ Toxicity

**Figure 1:**
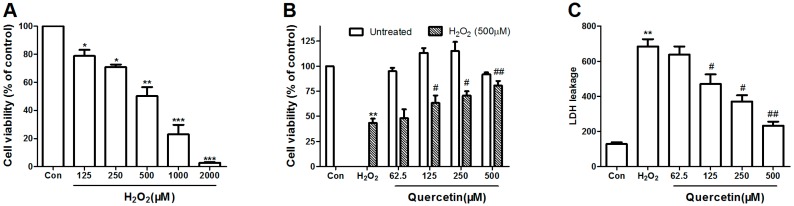
Protective effect of quercetin on H_2_O_2_-induced cytotoxicity in PC12 cells. (**A**) PC12 cells were treated with the indicated concentrations (125–2000 μM) of H_2_O_2_ for 24 h; (**B**) The cytotoxic of quercetin was examined after incubation with the indicated concentrations of quercetin for 24 h treatment using MTT assay. In the protected group, PC12 cells were pretreated with different concentrations of quercetin for 2 h, and then rinsed thrice with phosphate-buffered saline (PBS). Subsequently, the pretreated cells then were incubated with or without 500 μM H_2_O_2_ for an additional 24 h, and viability of cells was assessed by MTT assay. Percentage of cell viability was relative to the untreated control cells; (**C**) PC12 cells were pretreated with different concentrations of quercetin for 2 h, and then rinsed thrice with PBS. Subsequently, the pretreated cells then were incubated with or without 500 μM H_2_O_2_, after 24 h the supernatant was obtained for the measurements of LDH using commercial kits. Values represent mean ± SEM. of three independent experiments. * *p* < 0.05, ** *p* < 0.01, *** *p* < 0.001 versus control; ^#^ *p* < 0.05, ^##^ *p* < 0.01 versus H_2_O_2_ treated cells. LDH, lactate dehydrogenase; H_2_O_2_, hydrogen peroxide. Con, untreated.

To determine the IC50 dose of H_2_O_2_ toxicity on PC-12 cell, the viability was evaluated after 24 h exposure to different concentrations of H_2_O_2_ by the 3-[4,5-dimethylthiazol-2-yl]-2,5-diphenyl-tetrazolium bromide (MTT) method. Cell viability of PC-12 cells markedly decreased following incubation with H_2_O_2_ by a dose-dependent manner (125–2000 μM). As shown in Figure 1A, the IC50 value of H_2_O_2_ concentration was 500 μM (** *p* < 0.01), which resulted in 50% PC-12 cell inhibition. Thus, 500 μM H_2_O_2_ was used for the subsequent experiments to assess the protective effect of quercetin.

### 2.2. Protective Efficacy of Quercetin in H_2_O_2_-Induced Cytotoxicity

In order to determine the concentrations of quercetin which has nontoxic to cells, but could prevent H_2_O_2_-induced oxidative damage, we examined the cell viability in PC-12 cells after incubation with indicated concentrations quercetin. As shown in Figure 1B, quercetin had no cytotoxic effect in PC-12 cells at the concentrations up to 500 μM after 24 h treatment (*p* > 0.05).

To evaluation the protective efficacy of quercetin in H_2_O_2_-induced cytotoxicity, PC-12 cells were first pretreated for 2 h with the indicated concentrations of quercetin, and then rinsed thrice with PBS. Subsequently, the pretreated cells then were treated with 500 μM H_2_O_2_ for another 24 h. Results of the MTT assay following quercetin pretreatment showed a significantly increased cell viability compared to cells treated with H_2_O_2_ alone, in a dose-dependent manner. Pretreatment with 62.5, 125, 250 and 500 μM of quercetin increased the cell viability to 48.12 ± 3.52, 59.81 ± 2.78, and 70.12 ± 2.58 and 79.11 ± 1.643, respectively (Figure 1B).

To further explore the protective effect of quercetin, we examined the release of LDH, an indicator of cell injury, using the LDH cytotoxicity assay kit according to the manufacturer’s protocol. Pretreatment with quercetin reduced LDH release in a dose-dependent manner compared to cells treated with H_2_O_2_ alone (Figure 1C).

### 2.3. Quercetin Inhibits H_2_O_2_-Induced ROS and MDA Production

**Figure 2:**
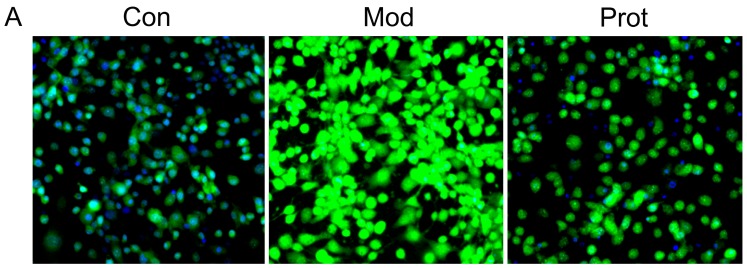

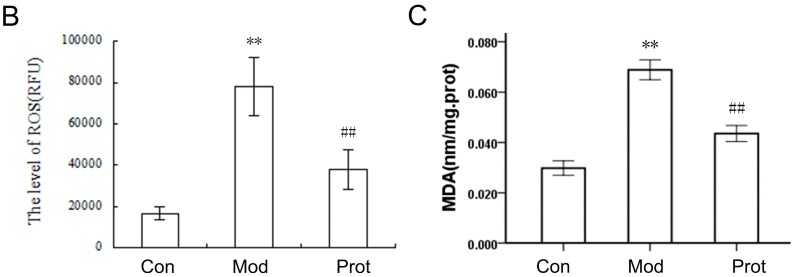
Quercetin inhibits H_2_O_2_-induced ROS and MDA production in PC-12 cells. (**A**) Cells were pretreated with quercetin (500 μM) for 2 h, and then rinsed thrice with PBS. Subsequently, the pretreated cells then were incubated with 500 μM H_2_O_2_ for another 24 h. Intracellular ROS levels were indicated by DCF fluorescence intensity and detected by high content screening; (**B**) the fluorescence intensity of group A were calculated, and the results were expressed as areas under the curve of relative light units (RLU); (**C**) cells were pretreated with quercetin (500 μM) for 2 h, and then rinsed thrice with PBS. Subsequently, the pretreated cells then were incubated with 500 μM H2O2 for another 24 h. The intracellular MDA levels were measured. Values represent means ± SEM, *n* = 3. ** *p* < 0.01 vs. control; ^##^ *p* < 0.01, vs. model. MDA, malondialdehyde; Con, untreated; Mod, H_2_O_2_ treated; Prot, quercetin + H_2_O_2_ treated.

Intracellular ROS by H_2_O_2_-induced was monitored by DCFH-DA in PC-12 cells using high content screening. As demonstrated in Figure 2A,B, incubation with H_2_O_2_ for 24 h led to an increase in DCF fluorescence intensity, which is proportionate to the amount of ROS generated. The result indicated that treatment with H_2_O_2_ increased intracellular ROS production in PC-12 cells (Model panel, Figure 2A) compared with the untreated cells (Control panel, Figure 2A). However, pretreatment with quercetin effectively reduced H_2_O_2_-induced ROS generation, as evidenced by the lower DCF fluorescence intensity in quercetin pretreated cells (Protect panel, Figure 2A).

MDA, which is the end product of lipoperoxidation and is considered to be a reliable indicator of oxidative stress [26]. The MDA level was measured using an MDA assay kit to investigate the effect of quercetin on H_2_O_2_-induced oxidative stress. A markedly elevation of the MDA level was observed in cells treated with H_2_O_2_ compared with the control group, whereas pretreatment with quercetin exhibited a statistically significant decrease in lipid peroxidation (Figure 2C). The results indicated that pretreatment of cells with quercetin inhibited H_2_O_2_-induced ROS and MDA production, alleviated lipoperoxidation of the cell membrane, and prevented cell damage.

### 2.4. Effects of Quercetin on H_2_O_2_-Induced Apoptosis

**Figure 3:**
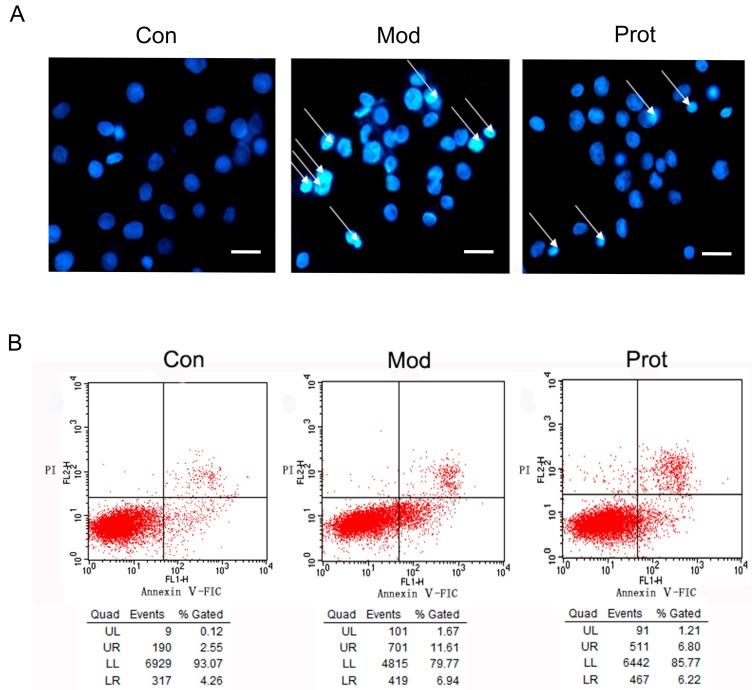
Effects of quercetin on H_2_O_2_ induced apoptosis. (**A**) The effect of quercetin on H_2_O_2_-induced apoptosis by Hoechst 33342 staining. Cells were pretreated with quercetin (500 μM) for 2 h, and then rinsed thrice with PBS. Subsequently, the pretreated cells then were incubated with 500 μM H_2_O_2_ for another 24 h, and were observed by fluorescence microscopy (200×) after nuclei staining with Hoechst 33342. The figures are representative for three different experiments; and (**B**) representative flow cytometry scatterplots of PI (*Y*-axis) vs. Annexin V-FITC (*x*-axis). Cells were pretreated with quercetin (500 μM) for 2 h, and then rinsed thrice with PBS. Subsequently, the pretreated cells then were incubated with 500 μM H_2_O_2_ for another 24 h, and were immediately analyzed using Annexin-V-FITC apoptosis detection kit by flow cytometry. Con, untreated; Mod, H_2_O_2_ treated; Prot, quercetin + H_2_O_2_ treated.

The oxidative stress induced by H_2_O_2_ is a crucial feature to promote cell death, we hypothesized that the anti-oxidative properties of quercetin may contribute to the protection of PC-12 cells from H_2_O_2_ induced apoptosis. To determine whether quercetin may attenuate cell apoptosis through reducing oxidative stress, we detected apoptotic morphological characteristics with Hoechst 33342 staining. PC-12 cells in the control group showed regular and round nuclei, as observed under the microscope (Control panel, Figure 3A). After exposure to H_2_O_2_ for 24 h, nuclei of PC-12 cells were observed with condensation and fragmentation, which was recognized as characteristic of apoptotic cells (Model panel, Figure 3A). Furthermore, pretreatment with quercetin significantly alleviated the effect of H_2_O_2_ induced apoptosis (Protect panel, Figure 3A).The phosphatidylserine expression on the cell membrane, an indicator of apoptotic cells, was also determined using Annexin V/PI staining and flow cytometry analysis. H_2_O_2_ exposure increased the apoptosis rate of PC-12 cells from 6.81 to 18.45% compared to the control, which was declined to 13.02% by 500 μM quercetin pretreatment (Figure 3B).

**Figure 4:**
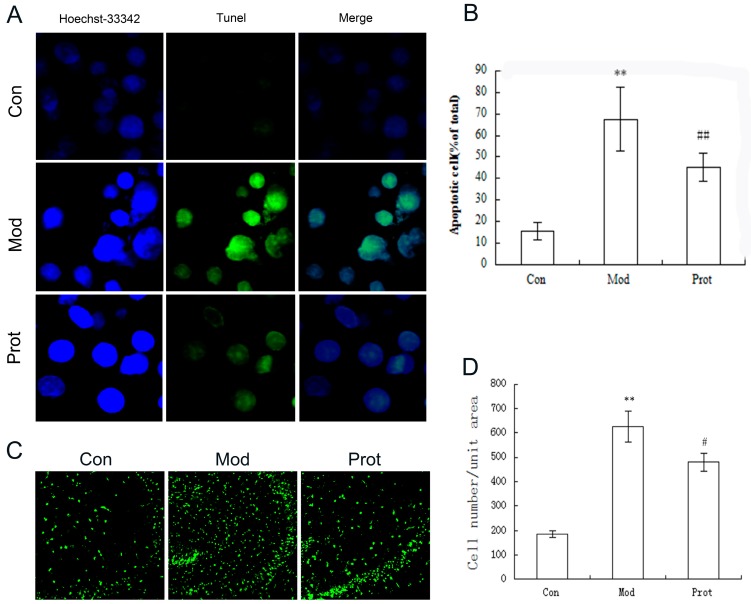
Protective effect of quercetin on H_2_O_2_ induced apoptosis in PC-12 cells and organotypic hippocampal slice were evaluated by TUNEL assay. (**A**) Representative images of TUNEL assay in PC-12 cells (400×); (**B**) quantification of TUNEL-positive cells in A; data collected from 10 fields for each group; experiments were repeated three times; (**C**) representative images of TUNEL assay in organotypic hippocampal slice (100×); and (**D**) quantification of TUNEL-positive cells in (**C**); data collected from 10 fields for each group; experiments were repeated three times. Data are expressed as overall mean ± SD of the ratio of apoptotic cells to the total number of cells. Statistical significance was determined using the Student’s *t*-test (** *p* < 0.01vs. control; ^##^ *p* < 0.01, ^#^ *p* < 0.05 vs. model). Con, untreated; Mod, H_2_O_2_ treated; Prot, quercetin + H_2_O_2_ treated.

To further investigate protective effect of quercetin on H_2_O_2_ induced apoptosis in PC-12 cells and organotypic hippocampal slices, a TUNEL (Terminal deoxynucleotidyl transferase mediated dUTP Nick End Labeling) assay was performed. As shown in Figure 4, 24 h incubation with H_2_O_2_ significantly increased the percentage of apoptotic PC-12 cells and hippocampal neurons compared with control group. However, the ratio of TUNEL-positive apoptotic cells decreased after pretreatment with quercetin. The result suggested that quercetin pretreatment markedly protected the cell death of PC-12 cells and hippocampal neurons by decreasing the apoptosis rate.

### 2.5. Effects of Quercetin on Antioxidant Enzyme Expression in H_2_O_2_-Induced PC-12 Cells

We hypothesized that the protective effects of quercetin against oxidative stress may involve the regulation of the expression of antioxidant enzyme, such as CAT, GSH-Px, and SOD, which are important antioxidant enzymes, under different conditions. As shown in Table 1, the expression levels of CAT, GSH-Px, and SOD were significantly higher in the control samples and lower in samples incubated with H_2_O_2_ alone. The CAT, GSH-Px, and SOD enzyme levels were significantly higher in quercetin pretreated samples compared to those incubated with H_2_O_2_ alone. These results demonstrated that H_2_O_2_ significantly inhibited the levels of antioxidant enzymes, but pretreatment with quercetin significantly attenuated this inhibition effect.

### 2.6. Effects of Quercetin on Expression of Apoptotic Proteins in H_2_O_2_-Induced PC-12 Cells

**Figure 5:**
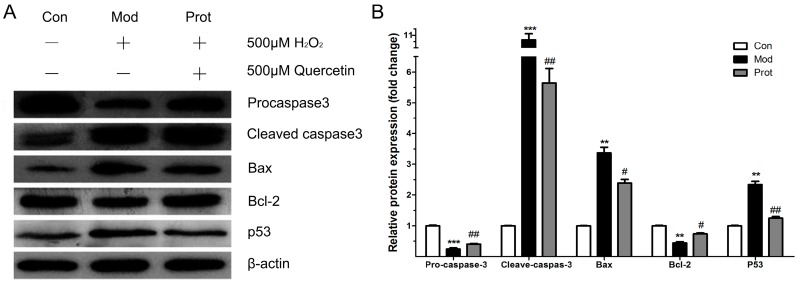
Effects of quercetin on expression of apoptotic proteins in H_2_O_2_-induced PC12 cells. (**A**) Western blot analysis of the expression of p53, Bcl-2, Bax, procaspase-3, cleaved caspase-3; and (**B**) the band intensities were measured with the Image J program, and shows the relative protein expression of quercetin on the expression of apoptotic proteins (*n* = 3). ** *p* < 0.01, *** *p* < 0.001 vs. control; ^##^ *p* < 0.01, ^#^ *p* < 0.05 vs. model. Con, untreated; Mod, H_2_O_2_ treated; Prot, quercetin + H_2_O_2_ treated.

To investigate the neuroprotective mechanism of quercetin on H_2_O_2_-induced apoptosis, the expression of p53, Bcl-2, Bax, cleaved caspase-3, and procaspase-3 were evaluated by Western blotting. Figure 5 shows the protein expression of p53, Bcl-2, Bax, procaspase-3, cleaved caspase-3, and β-actin after 24 h with or without quercetin pretreatment in PC-12 cells exposed to 500 μM H_2_O_2_. The pro-apoptotic protein Bax was significantly increased, while the anti-apoptotic protein Bcl-2 was decreased by H_2_O_2_ treatment. Pretreatment with quercetin significantly increased Bcl-2 and decreased Bax expressions. These results suggested that quercetin inhibited H_2_O_2_ induced apoptosis by decreasing the ratio of Bax/Bcl-2. The changes of Bax/Bcl-2 levels implicated in the initiation of caspase signaling, we investigated the caspase-3 and p53 activation in this study. As shown in Figure 5, compared to the control group, H_2_O_2_ induced an obviously decreased procaspase-3 and an increased cleaved caspase-3 and p53. However, quercetin revealed enhanced procaspase-3 activity and reduced cleaved caspase-3 and p53 expression compared with the H_2_O_2_ groups. These results suggested that quercetin decreased cleaved caspase-3 and p53 expression to inhibit H_2_O_2_-induced apoptosis.

## 3. Discussion

**Figure 6:**
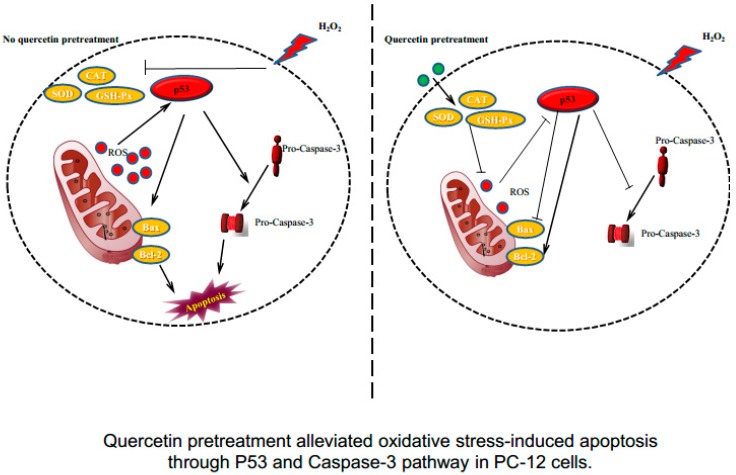
Quercetin pretreatment alleviated oxidative stress-induced apoptosis through the P53 and caspase-3 pathways in PC-12 cells.

In the present study, the protective efficacy of quercetin on H_2_O_2_-induced sustained oxidative stress was evaluated in PC-12 cells. The present findings demonstrated that quercetin pretreatment remarkably increased cell viability and reduced LDH release compared to cells treated with H_2_O_2_ alone in a dose-dependent manner. Furthermore, we observed that quercetin pretreatment significantly reduced intracellular ROS levels and MDA production, and attenuated cell apoptosis in a model of H_2_O_2_-induced oxidative stress. In addition, quercetin pretreatment obviously increases antioxidant enzyme expression and upregulates Bcl-2, downregulates Bax, caspase-3, and p53 activation. This present study demonstrates that quercetin markedly protects against H_2_O_2_-induced oxidative stress and cell apoptosis in PC-12 cells (Figure 6).

A growing number of studies have demonstrated that oxidative damage from ROS is widely implicated in neurodegenerative disorders [5,6]. In our study, treatment with 500 μM H_2_O_2_ for 24 h was used to induce sustained oxidative stress, which significantly caused a dose-dependent loss of cell viability in PC-12 cells. However, appropriate concentrations (62.5, 125, 250 or 500 μM) of quercetin pretreatment significantly reduced H_2_O_2_-induced loss of cell viability, which was affirmed by the attenuation in LDH release (Figure 1). The results showed that pretreatment with quercetin attenuated the H_2_O_2_-induced release of LDH, affirming the protective role of quercetin in H_2_O_2_-induced cytotoxicity. We further investigate protective effect of quercetin on H_2_O_2_ induced apoptosis using Hoechst 33342 staining, Annexin V/PI staining, and flow cytometry analysis. Pretreatment with quercetin significantly alleviated the effect of H_2_O_2_-induced apoptosis, condensation, and fragmentation of cell nuclei (Figure 3).

To confirm the protective effectiveness of quercetin on H_2_O_2_-induced oxidative stress, further studies and evaluations using organotypic hippocampal slice were performed. The cell lines have altered intrinsic physiology, modified proliferation, and a changed life cycle, which is not in accord with physiological conditions. Hippocampal slice culture is widely used as a simplified, advantageous system in vitro for studying regeneration and neurodegeneration [27]. Organotypic hippocampal slice culture is similar to in vivo models, offering the possibility of analyzing neurogenesis and neuronal circuits, and is simpler compared to the neocortex [28]. In this study, we investigate the protective effect of quercetin on H_2_O_2_-induced apoptosis in PC-12 cells and organotypic hippocampal slice using TUNEL assay. The results suggested that quercetin pretreatment markedly reduced the loss of PC-12 cells and hippocampal neurons possibly through a decrease in apoptosis (Figure 4).

Previous studies demonstrated that neuronal cells are more susceptible to oxidative stress in comparison to other cell types because of poor antioxidant defenses [29]. To further test the protective effectiveness of quercetin on H_2_O_2_-induced oxidative stress, we monitored intracellular ROS in PC-12 cells by DCFH-DA using high content screening. H_2_O_2_-induced ROS generation was effectively reduced by quercetin pretreatment, indicating that it would be effective against downstream targets of ROS which are responsible for neuronal death. We also measured the MDA level, which is a naturally-occurring end product of lipid peroxidation and is a biomarker of oxidative stress [26]. Suppressing or reversing the progression of lipid peroxidation exhibited the protective properties of antioxidant molecules. Pretreatment with quercetin exhibited a statistically significant decrease in lipid peroxidation (Figure 2C). These results indicated that quercetin pretreatment inhibited H_2_O_2_-induced ROS production and alleviated lipoperoxidation of cell membranes, in accordance with an inhibition of oxidative stress and prevention of cell damage. The result is consistent with previous studies, which have also affirmed the protective effects of quercetin against oxidative stress [21,30].

The inhibition of H_2_O_2_-induced intracellular ROS generation and cell lipid peroxidation by quercetin pretreatment may occur via a direct antioxidant mechanism through free radical-scavenging activity. To validate the neuroprotective role and antioxidant capability of quercetin, we measured the levels of endogenous antioxidant enzymes, including catalase (CAT), superoxide dismutase (SOD), and glutathione peroxidase (GSH-Px). SOD is a vital enzyme that regulates oxidative stress, which catalyzes the dismutation of the superoxide anion to oxygen molecules and hydrogen peroxide [31]. Catalase plays an important role in eliminating hydrogen peroxide through converting hydrogen peroxide to water and oxygen [32,33]. Previous study found that the ROS and SOD levels showed no significant change in the rat HC region of rat brains treated with quercetin as compared to rats in the control group [21]. The SOD and CAT activity in the H_2_O_2_-treated group were significantly reduced compared to the control group. Pretreatment with quercetin significantly increased and restored SOD and CAT levels, which may protect against H_2_O_2_-induced oxidative damage. GSH-Px is a vital antioxidant enzyme that catalyzes the reduction of hydroperoxides at the expense of reduced GSH, which is largely identified in the cytoplasm and mitochondria compartment of eukaryotic cells [34,35]. In this study, the GSH-Px level was significantly increased after quercetin pretreatment compared to H_2_O_2_-treated PC-12 cells alone. The increasing levels of endogenous antioxidant enzymes, in turn, catalyzed the detoxification of superoxide radicals to less toxic molecules, led to the attenuation of oxidative stress, and is directly correlated with increased cellular antioxidant capabilities [36,37]. These results suggest that quercetin could activate the native antioxidant mechanisms in the PC-12 cells during the pretreatment phase, which was able to protect these cells from undergoing H_2_O_2_-induced oxidative damage.

Previous studies have demonstrated that H_2_O_2_ regulated the balance of between Bcl-2 and Bax, altered mitochondrial membrane permeability, activated caspase cascades, and upregulated p53 expression, subsequently leading to apoptosis [38,39]. The Bcl-2 family members play an important role in apoptosis and is associated to cellular injury during chronic neurodegenerative disorders [40]. Previous study found that the protein levels of Bax, Bcl-2, cyt-c, cleaved caspase-3, and p53 were not significantly changed in the rat HC region of rat brains treated with quercetin as compared to rats in the control group [21]. In the present study, we found that quercetin pretreatment reversed the alternations of Bax and Bcl-2 expressions induced by H_2_O_2_, and substantially restored the balance of Bcl-2/Bax. The cascading activation of caspases plays an important role in apoptosis, which is the central component of apoptotic pathways [41,42]. The caspase activation during ROS exposure has been closely implicated in the pathogenesis of neurodegenerative disorders, such as AD [43]. Caspase-3 is a potential effector of apoptosis, which is triggered via several different pathways. In this study, we demonstrate that pretreatment with quercetin significantly decreases apoptosis and caspase-3 activity in PC-12 cells exposed to H_2_O_2_. The tumor suppressor p53 is an important activator of the ROS-mediated apoptotic pathway [21,38]. Our results demonstrate that quercetin pretreatment attenuated the expression of p53 induced by H_2_O_2_ in PC-12 cells. Previous studies show that p53 differentially regulated Bcl-2 and Bax levels both in vitro and in vivo [44]. Our results are in agreement with previous studies which have shown that quercetin increased Bcl-2 and decreased Bax expression concomitant with decreased p53 expression. The result suggested that the neuroprotective effects of quercetin on H_2_O_2_-induced apoptosis in PC-12 cells may be mediated via the p53 pathway, and further studies are warranted (Figure 6).

## 4. Materials and Methods

### 4.1. Chemical, Reagents, and Antibodies

Quercetin was purchased from Aladdin chemistry Co. Ltd. (Shanghai, China) and was dissolved in dimethyl sulphoxide (DMSO) to prepare the stock solution of 100 mM. Millicell-CM was purchased from Millipore (Billerica, MA, USA). Fetal calf serum (FCS) and Dulbecco’s modified eagle’s medium (DMEM) were purchased from Gibco (Gaithersburg, MD, USA). 3-[4,5-dimethylthiazol-2-yl]-2,5-diphenyl-tetrazolium bromide (MTT), H_2_O_2_, 2′,7′-dichlorofluorescein diacetate (DCFH-DA), and Hoechst 33342 were purchased from Sigma (St Louis, MO, USA). The kits for MDA, LDH, CAT, SOD, and GSH-Px were obtained from Jiancheng Bioengineering Institute (Nanjing, China). DeadEnd™ Fluorometric TUNEL system was purchased from Promega (Madison, WI, USA). An Annexin-V-FITC apoptosis detection kit was purchased from BD Biosciences (Franklin Lakes, NJ, USA). Antibodies against caspase-3, p53, Bax, and Bcl-2 were purchased from Cell Signaling Technology (Danvers, MA, USA). Antibodies against β-actin and HRP conjugated anti-rabbit IgG were purchased from Zhong Shan Golden Bridge Biological Technology Co. Ltd. (Beijing, China). Streptomycin and penicillin were obtained from Invitrogen (Carlsbad, CA, USA). Other general agents were purchased from commercial suppliers.

### 4.2. Cell Culture

PC-12 cells were obtained from Shanghai Institute of biological Science, Chinese Academy of Science, and were routinely cultured in DMEM supplemented with 10% FCS, 100 U/mL penicillin, and 100 μg/mL streptomycin in a humidified incubator (5% CO_2_ at 37 °C).

### 4.3. PC-12 Cell Pretreatment and Induction of Oxidative Stress

PC-12 cells were cultured in 96-well culture plates at a density of 1 × 10^4^ cells/per well, and cultured with 5% CO_2_ at 37 °C for 24 h. Cells at approximately 70% confluence were pretreated with 62.5, 125, 250 or 500 μM quercetin in serum-free DMEM at 37 °C for 2 h, and then rinsed thrice with PBS. Subsequently, the pretreated cells were exposed to 500 μM H_2_O_2_ in serum-free DMEM for another 24 h. Untreated cells and cells treated with H_2_O_2_ alone were used as normal and H_2_O_2_ control, respectively.

### 4.4. Organotypic Hippocampal Slice Cultures

C57BL/6J mice were used for organotypic hippocampal slice. The mice were bred at the Experimental Animal Center of Henan University, and all experimental disposals were performed in accordance with the Guidance Suggestions for the Care and Use of Laboratory Animals by Ministry of Science and Technology of the People’s Republic of China. Hippocampal slice cultures with a thickness of 350 nm were prepared at P7 (postnatal day 7) as described before [45,46]. The organotypic hippocampal slices were cultivated on Millicell-CM in six-well plate in a humidified incubator (5% CO_2_ at 37 °C). Each well was filled with 1.2 mL incubation medium and was changed every second day.

### 4.5. MTT Assay of Cell Viability

Cellular toxicities of H_2_O_2_ and quercetin were determined in PC-12 cells using the MTT assay. PC-12 cells were cultured in 96-well culture plates at a density of 1 × 10^4^ cells/per well, and cultured with 5% CO_2_ at 37 °C for 24 h. After the indicated concentrations of H_2_O_2_ or quercetin, cell viability was determined by MTT assay. Control groups consisted of cells incubated with medium only. After appropriate time intervals, the medium was removed and replaced by 200 μL growth medium with 0.5 mg/mL MTT, and the plates were incubated for an additional 4 h at 37 °C. Subsequently, the supernatant was removed and replaced by 150 μL DMSO to dissolve the formazan crystals. The optical density (OD) was measured at a 570 nm using a 96-well multiscanner autoreader (Bio-Rad, Hercules, CA, USA). The results were presented as a percentage of the control.

### 4.6. Determination of Intracellular ROS

Intracellular ROS levels were examined using a non-fluorescent agent DCFH-DA. DCFH-DA is a non-fluorescent lipophilic ester that easily penetrates the plasma membrane and passes into the cytosol, where this molecule was activated by esterase-mediated cleavage of acetate to form DCFH. DCFH was oxidized to the formation of DCF in the presence of ROS, results in a green fluorescence. The fluorescence intensity is generally considered to reflect the level of intracellular ROS [47]. In brief, PC-12 cells (5 × l0^3^ cells/well) were seeded in 96-well plates for 24 h incubation. After the indicated treatment, cells were washed with PBS buffer and were incubated with DCFH-DA in complete medium at 37 °C for 30 min. After incubation, cells were washed with PBS and the intracellular ROS was also detected by observing DCF using high-content screening (HCS, Thermo Fisher Scientific, Waltham, MA, USA) at an excitation wavelength of 485 nm and an emission wavelength of 525 nm, respectively. Control groups consisted of cells incubated with medium only.

### 4.7. Hoechst 33342 Staining and FACS Assay with Annexin V/PI Staining

We assessed apoptosis using Hoechst 33342 and fluorescence activated cell sorting (FACS) analysis. In Hoechst 33342 staining, PC-12 cells were seeded into six-well plates, after various treatment cells were stained with 1 μg/mL Hoechst 33342 for 5 min. The cells were washed and fixed with 4% paraformaldehyde in PBS for 5 min at 4 °C, then observed by fluorescence microscopy. Cells with condensed nuclei were considered apoptotic, and the percentage of apoptotic cells in PC-12 was determined by examining at least 300 cells per group, at ×400 magnification. The apoptosis assays were also conducted using an Annexin-V-FITC apoptosis detection kit by flow cytometry, according to the manufacturer’s instructions. PC-12 cells were exposed to various conditions treatment, and were harvested and washed twice with cold PBS, resuspended in 1× binding buffer. After incubation with Annexin V for 15 min in the dark at RT, the cells were immediately analyzed by flow cytometry.

*TUNEL assay:* PC-12 cells were seeded on coverslips of six-well plates for 24 h incubation, and treated with the indicated conditions. TUNEL assay was performed according to the manufacturer’s instructions to assessed apoptosis of various groups. Apoptotic cells were observed by a fluorescent microscope (BX51, OLYMPUS, Tokyo, Japan) at an excitation wavelength of 515–565 nm.

After the indicated treatment, organotypic hippocampal slice cultures were fixed with 4% paraformaldehyde in PBS for 30 min. TUNEL assay was performed according to the manufacturer’s instructions. At least five randomly-chosen areas in every slide were used. Percent apoptosis was determined by counting the number of apoptotic cells and dividing by the total number of cells in the areas.

### 4.8. Biochemical Analysis

Each group sample of treated and untreated PC-12 cells was collected using cold PBS by specific centrifugation condition required by each enzyme kit. The cells were homogenized using cold-PBS and the supernatant was obtained for the measurements of malondialdehyde (MDA), LDH, CAT, GSH-Px, and SOD activities using commercial kits.

### 4.9. Western Blot Analysis

After the treatments as described above, the expression levels of procaspase-3, cleaved caspase-3, Bax, Bcl-2, and p53 were analyzed with Western blotting. Briefly, cells were washed twice with PBS and lysed in ice-cold RIPA, and the protein concentrations of the supernatants were determined using the BCA protein assay kit. Equal amounts of proteins were separated by 12% SDS-PAGE and transferred onto polyvinylidene difluoride (PVDF) membranes. Membranes were blocked with 5% non-fat milk in TBST for 1 h at room temperature and then incubated with primary antibodies against procaspase-3, cleaved caspase-3, Bcl-2, Bax, p53, or β-actin at 4 °C overnight. On the second day, the membranes were washed and incubated with rabbit or mouse IgG conjugated to horseradish peroxidase for 2 h, then washed with TBST buffer three times and the proteins were detected using enhanced chemiluminescene substrate (ECL, Beyotime, Shanghai, China). The expression level of target proteins relative to β-actin used a semi-quantitative comparison between the different experimental groups.

### 4.10. Statistical Analysis

All experiments were repeated three times in triplicate. Results are presented as the mean ± standard deviation (SD). Data were analyzed using Image J (NIH, Bethesda, MD, USA) and SPSS Inc. software (SPSS Statistics, V19.0.0, IBM, Armonk, NY, USA). Statistical significance was calculated using the *t*-test for paired samples. *p* < 0.05 was considered as significant in statistically, and *p* < 0.01 as highly significant.

## 5. Conclusions

In this study, our findings confirm that quercetin pretreatment significantly inhibited H_2_O_2_-induced ROS and MDA production and alleviated lipoperoxidation of cell membranes and prevented cell damage. Furthermore, quercetin pretreatment markedly reduced the apoptosis of PC-12 cells and hippocampal neurons, attenuating this inhibition effect of the antioxidant enzymes CAT, GSH-Px, and SOD in PC-12 cells exposed to H_2_O_2_. In addition, pretreatment with quercetin significantly increased Bcl-2 and decreased Bax expressions, while reducing cleaved caspase-3 and p53 expression. In conclusion, this study demonstrated that quercetin protects PC-12 neuronal cells against H_2_O_2_-induced oxidative stress and elucidates several underlying mechanisms. These findings support the further investigation of quercetin as a new therapeutic agent in the treatment of oxidative stress in neurodegenerative diseases.

## Figures and Tables

**Table 1:** Effects of quercetin on antioxidant enzyme expression in H2O2-induced PC12 cells.

Group	CAT (U/mg Prot)	SOD (U/mg Prot)	GSH-Px (U/mg Prot)
**Con**	0.695 ± 0.035	35.537 ± 2.230	5.692 ± 0.202
**Mod**	0.265 ± 0.021 **	14.276 ± 0.813 **	3.708 ± 0.130 *
**Prot**	1.500 ± 0.057 ^##^	27.000 ± 1.397 ^##^	4.734 ± 0.157 ^#^

** *p* < 0.01, * *p* < 0.05 vs con; ^##^ *p* < 0.01, ^#^ *p* < 0.05 vs. mod. CAT, catalase; SOD, superoxide dismutase; GSH-Px, glutathione peroxidase. Con, untreated; Mod, treated; Prot, quercetin + H_2_O_2_ treated.
